# Effective trialing of digital interventions in daily life – the mental health perspective

**DOI:** 10.1038/s41746-026-03414-7

**Published:** 2026-11-06

**Authors:** Ulrich W. Ebner-Priemer, Lorenz Deserno, Markus Reichert

**Affiliations:** 1 Mental mHealth Lab, Department of Sports and Sports Science, Karlsruhe Institute of Technology, Karlsruhe, Germany; 2 Department of Psychiatry and Psychotherapy, Central Institute of Mental Health, Medical Faculty Mannheim, Heidelberg University, Mannheim, Germany; 3 German Center for Mental Health, Partner Site Mannheim, Mannheim, Germany; 4 Center of Mental Health, Department for Child and Adolescent Psychiatry, Psychosomatics and Psychotherapy, University Hospital of Würzburg, Würzburg, Germany; 5 Department of Sport and Exercise Science, Faculty of Natural and Life Sciences, University Salzburg, Salzburg, Austria

**Keywords:** Diseases, Health care, Psychology, Psychology

## Abstract

Digital mental health interventions show promise but have yet to meet expectations. We discuss gaps between promising results of pilot studies and null findings in randomized controlled trials, focusing on two major approaches: just-in-time relapse prevention and just-in-time adaptive interventions. We propose that relapse prevention will profit from long-term studies capturing emerging episodes, and we suggest a novel framework for adaptive interventions including causality testing to proceed towards effective trialing.

## Introduction

The WHO expects digital interventions to address current healthcare challenges and to transform health prevention and treatment^1^. For mental health, digital interventions reveal opportunities beyond scalability. First, mental health conditions often include harmful behavior as core classification criteria and starting point for interventions, such as alcohol consumption or avoidance behavior. Behavioral techniques, delivered via digital interventions^2^, can therefore directly address mental health. Second, symptoms of mental disorders are context-dependent. They often emerge in specific contexts and fluctuate over time; therefore, symptoms should be assessed and treated in relevant environmental contexts^3^. For example, ‘morning depression’ can best be captured at a certain time, and anxiety can be reduced in a safe therapy environment.

**Fig. 1:**
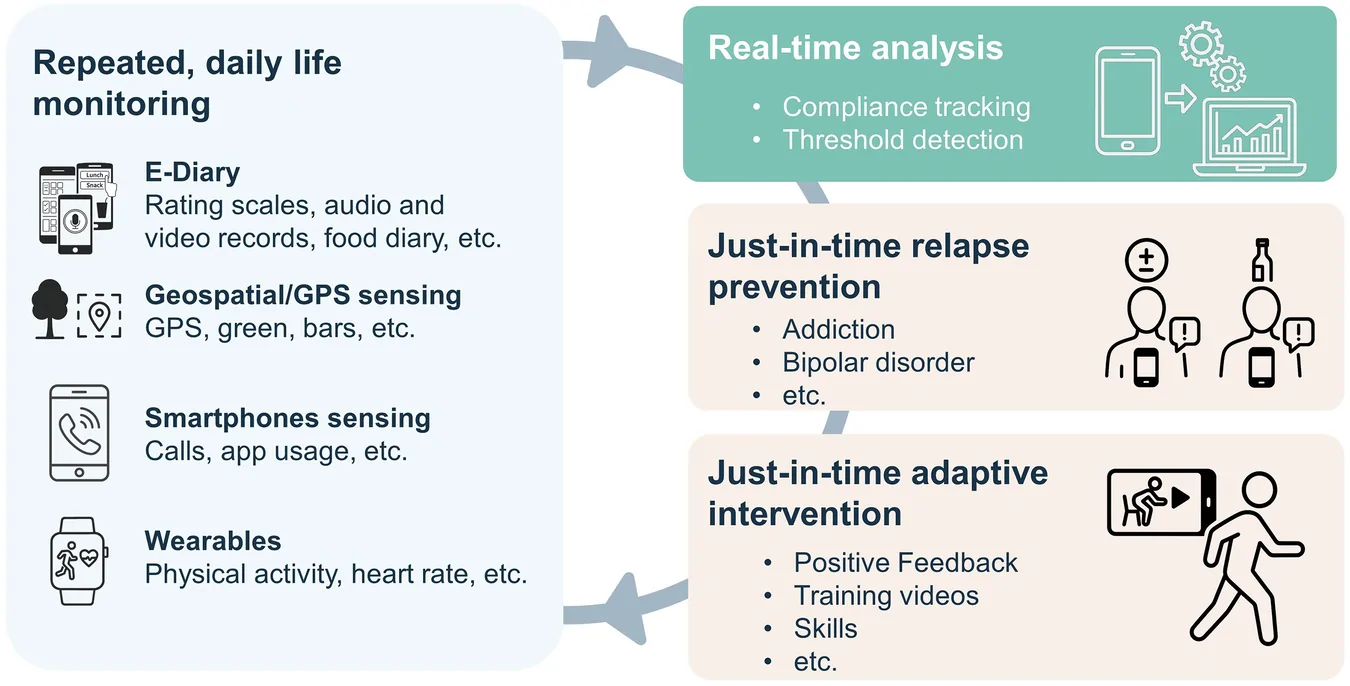
Illustration of the “mHealth toolbox”. This “toolbox” summarizes the possibilities promised by digital interventions. This includes repeated daily life monitoring in real time (left-hand side blue box) through e-diaries, geospatial factors, smartphone sensing and wearables, as well as real-time analyses (right-hand side, upper green box) and associated just-in-time relapse prevention (right-hand side, middle beige box) and/or just-in-time adaptive intervention (right-hand side, lower beige box).

Fortunately, mental mobile Health (mental mHealth) interventions have been tremendously benefitted by technological progress across the last decades. Figure 1 illustrates the key methodological environment for mental mHealth interventions, which we call “mHealth toolbox”. Symptoms and contexts relevant for putative interventions can be tracked in daily life using smartphones and wearables and, importantly, they can be analyzed in real-time. The mHealth toolbox comes with two unique chances: First, there is just-in-time relapse prevention, whereby situations of high mental health risk can be detected early, such as emerging depressive or manic episodes. External help can then be delivered in time, e.g., adaptations in pharmacological treatment, to prevent new episodes of illness^4^. Second, there are just-in-time adaptive interventions, which trigger micro-treatments such as mindfulness interventions to decrease momentary craving for alcohol, after detecting moments when participants might be especially susceptible to behavior change techniques. Just-in-time relapse prevention and just-in-time adaptive interventions draw from the mHealth toolbox and make use of repeated, daily life monitoring and real-time analysis. However, they are aiming for distinct goals. Relapse prevention strives to detect upcoming episodes of mental disorders, preventing them with traditional pharmacological or psychotherapeutic treatment. Just-in-time adaptive interventions, on the other hand, are digital real-life methods designed to motivate behavior that reduces psychopathological symptoms or protects mental health. In addition, real-time mHealth approaches can be used for preventing episodes and promoting resilience. For example, in healthy individuals, resilience may be improved using stress training. Similarly, individuals transitioning to a first episode of depression^5^ or individuals with “*clinical high risk*” of transitioning to a first episode of psychosis may benefit from real-time mHealth approaches^6^. Although we do not address these applications in detail, the discussed backbone may inform the development of real-time mHealth approaches for resilience/promotive interventions, too.

Although methodological possibilities are appealing and despite the proliferation of commercial mental health apps, progress in both areas, just-in-time prevention and intervention, remains limited, and most commercial apps lack scientific validation^7,8^ and rigorous scientific testing^9^. Here we ask why mental mHealth has not yet lived up to expectations^10,11^. We discuss how to translate methodological advances and promising observational findings into effective trialing of digital mental health interventions. Towards this aim, we first highlight prominent gaps between promising pilot data and failing RCTs. Second, we expand on future steps essential for effective trialing for both just-in-time relapse prevention and just-in-time adaptive intervention.

### Gaps between promising pilot data and failing RCTs

Promising pilot studies often do not translate into successful RCT outcomes^12,13^. In our view, reasons are rooted in the implementation of the digital interventions, as well as in overestimated expectations caused by alpha-inflation and temporal data leakage.

First, pleasant myths about digital interventions persist, e.g., stating that mobile applications “*are cool and engaging, so patients will love to use them*”^11^. However, both EMIs (ecological momentary interventions) and JITAIs (just-in-time adaptive interventions) are burdensome in *implementation and use*. A recent meta-analysis^14^ on smartphone delivered interventions investigating adherence and attrition across 70 RCTs reported that only 34% of users completed the full intervention. This is in line with industry market data, which shows that almost 75% of users quit the use of health apps after only ten logins^15^. Without specific measures, missing data (resp. missing use) of the digital intervention will be the norm. For example, real-time analyses systems, including compliance dashboards, and adaptive designs are helpful to boost compliance^16^. Adaptive designs come with a low number of prompts and pose reduced burden on most sampling days, but assessments increase on days of diagnostic uncertainty. For example, the GEMCO^17^ study uses a single item to track affective states on a daily level in patients with depression and bipolar disorders (BD) across 24 months. However, on days of diagnostic uncertainty linked to an increased chance of an emerging episode, additional items and even face-to-face interviews are automatically provided. Therewith, adaptive designs balance accuracy and patient burden in real-time.

Another myth in EMI^11^ is that “*all we need is the EMA/EMI app to address all problems of an individual patient, and we can reduce or remove the need for clinical involvement”*^11^. Empirical evidence has proven this assumption wrong and shows blended designs to be empirically advantageous. For example, the SELFIE-RCT^18^ testing a blended app-based, adaptive EMI with three face-to-face sessions revealed impressive effect sizes for improvements in self-esteem of youth suffering from childhood adversity^19^. Additionally, the inclusion of gamification elements (e.g., incentives, leaderboards, badges, community rankings) can benefit compliance and adherence. For example, the motivation to continue use of a digital intervention could be predicted by feelings of competence, autonomy, and relatedness induced by gamification elements in one study^20^. Still, there is a lack of systematic evidence on the effects of gamification elements on compliance and adherence to digital interventions; especially, it may be helpful to include the first-person perspective of individuals with lived experience to tailor gamification elements in future studies that tackle this proposal.

Second, *alpha-inflation* may explain why promising observational studies do not result in successful relapse prevention RCTs. Digital phenotyping provides numerous predictor-outcome combinations, so chance findings are inevitable; this inflates the risk of false positives. For example, the brute-force extraction of thousands of predefined voice features from mobile phone data (e.g., the 6,552-feature ‘affective set’^21,22^), generates hundreds of false positives when predicting relapse or emerging affective episodes. To account for this challenge, an integrative analytical strategy may be considered, such as combining multiple predictors into latent variables^22^. In addition, clinical relevance should be critically considered on both a conceptual and an empirical level, e.g., asking questions such as: Why are incoming phone calls very strong predictor of manic episodes, but not outgoing phone calls or other talkativeness-related digital phenotypes? In addition, sensitivity-specificity scatter plots^23^ or ROC curves^24^ might help to estimate the clinical relevance of findings. Towards just-in-time relapse prevention, they can convey clinically relevant indices yielding information, e.g., on the number of emerging manic episodes that can be detected and the number of false alarms to be expected.

Third, a phenomenon known as “*temporal data leakage*” in machine learning research^25^ may further contribute to the outlined gap between promising pilot data and null findings of RCTs. In short, temporal data leakage describes the unintentional use of future data to explain current states. For example, micro-longitudinal data are commonly analysed with multilevel models, also called hierarchical or random effects models. These employ subject-centring to disentangle within- and between-subject effects (see the paragraph on ecological fallacy^26^ later on for details). For well-established reasons, these methods are the gold-standard procedure for hypothesis testing in intensive longitudinal data research^27^. However, their use for just-in-time prediction can be problematic because they “train” the model with access to forthcoming symptomatology, including “future” data of the full monitoring period. In contrast, prospective RCTs will start without this information. To overcome this limitation, prospective algorithms are needed, such as Statistical Process Control methods^20^.

### Just-in-time relapse prevention

The potential of just-in-time relapse prevention becomes most face valid in BD. This disorder is episodic in nature; a main treatment target is the prevention of relapses, and the main symptomatology is easily accessible via smartphones (e.g., altered sleep, communication, and activity). Unfortunately, substantial inconsistencies and unreplicable findings are the norm rather than the exception in BD just-in-time relapse prevention trials²³. While observational studies report an accuracy rate of over 95% in predicting new illness episodes, all current RCTs using digital phenotyping to predict and prevent emerging episodes have failed⁶.

To fully leverage the potential of just-in-time relapse prevention, we recommend the future studies to: (i) *ensure sufficient variance in the outcome parameters*^10^; (ii) *increase temporal precision in the ground truth*; and (iii) *develop digital phenotypes with high validity*^28^.

(i) Ensure sufficient variance in the outcome parameters
  To discover predictors of new illness episodes, e.g., relapse in addiction or affective episodes in BD, studies are necessary which observe such transitions. This may sound trivial, but many studies fail to do so by focussing on too short assessment periods. A systematic investigation of observational digital phenotyping studies on emerging episodes in BD^22^ revealed that the most common study duration was 12 weeks. It is unrealistic to expect episode emergence within such a short timeframe. This problem persists to intervention studies. For example, the landmark studies MONARCA I and II^12,13^ implemented a total study period of six and nine months. The estimated chance of experiencing at least one upcoming illness episode was less than 20% per patient^10^. That this is not solely a problem in BD, is evidenced by studies with depression also limited by short total assessment periods and yielding disappointing results^29^. In the end, studies predicting dimensional measures of depression or using cross-sectional comparisons between healthy participants and patient populations, are not well suited to predict actual relapse or new episodes. They show low generalizability^22^ and therefore provide a weak foundation for later RCTs. Fortunately, more recent RCTs and observational studies have adopted longer durations, such as BipoLife^30^ (21 months), Recode^31^ (12 months), RADAR^32^ (12 months), and GEMCO^33^ (24 months).
  In sum, we argue that the probability of an emerging episode in a given time period must be taken into account when planning a study. As a rule of thumb, we recommend designs that provide a natural relapse probability of at least 50% within the study period. In our studies this roughly translates to periods above 12 months, but of course, it depends on illness severity of the sample under investigation.
(ii) Increase temporal precision in the ground truth
  The second major issue is the infrequent and imprecise tracking of outcomes. Put simple, not only new emerging episodes are needed to build powerful prediction models, but we have to precisely know when those episodes emerged to be able to build reliable predictive models. Monthly clinical ratings were the most common method used in digital phenotyping trials of BD to detect early warning signals^22^. Categorical expert ratings, such as structured clinical interviews^34^, usually cover a period of two weeks. Given one assessment per month, this would result in a classification status over two weeks (ill: yes or no) for 50% of the time, whereas the precise onset and severity of an upcoming episode remains unknown. Even worse are monthly dimensional interviews (e.g., the Montgomery-Åsberg Depression Rating Scale^35^) which typically cover just four days, leaving ~85% of the days without ground truth data. Such sparse data limits episode detection and undermines generalizable prediction models.
  A similar picture is evident in depression research. RADAR-MDD (Remote assessment of disease and relapse in MDD)^32^, the most impressive mHealth study in MDD, is a multisite prospective cohort study, monitoring 600 participants over 12 months, with a primary interest in feasibility and the question of which digital phenotypes are suitable as proxies for depressive symptomatology. Symptom severity – as ground truth – was only assessed every three months, resulting in too few detected upcoming episodes and making temporal predictions of upcoming episodes impossible^36^.
  In sum, to investigate prodromal symptomatology, the exact start of a new episode must be identifiable. Of course, it is impossible to determine the exact moment when a new episode begins (e.g. Monday at 15:30). However, as a rule of thumb, we recommend a design enabling the classification of each given week in a data set as either ill or euthymic.
(ii) Develop digital phenotypes with high validity
  In mental mHealth, researchers have often prioritized ease of data collection over construct validity^22^. Examples include digital phenotype predictors such as ‘number of unanswered incoming calls’, ‘Wi-Fi upload data’, or ‘Facebook app usage’. They are easy to measure, but weakly linked to diagnostic criteria, i.e., their construct validity is low. Accordingly, their utility in relapse prediction is small. What is needed, instead, are smart digital phenotypes^28^, such as the number of words spoken within a minute, which show higher validity to the criterion “increased talkativeness or pressured speech”^34^ to promise improved prediction.
  The unprecedented development in digital technology might bear possibilities beyond digital phenotyping. Cognitive-behavioral tasks may be an interesting example. Whereas earlier tasks were found to be rather unreliable and to generalize poorly in training contexts^37^, designing cognitive-behavioral tasks as games^38^, and in particular as small and relatively simple games (e.g. “candy crush saga”^39^) look promising. Indeed, such “cognitive games” tested in about 5 minutes reached moderate to good levels of between-subject test-retest reliability^40^. Furthermore, a recent study in a small sample of healthy individuals showed that real-life physiological signatures of reward-based learning (as measured by ECG and EEG) can predict upcoming fast and slow mood fluctuations^41^. Therefore, cognitive-behavioral tasks combined with physiological measurements show promise for the future.
  In sum, many studies observing associations between digital phenotypes and affective symptomatology are characterized by limited generalizability and validity. Not surprisingly, those findings did not translate into successful RCTs. What is needed to enable real-time relapse prevention are long-term studies investigating actual emerging episodes and a characterization of those episodes across time. Further, predictors of high validity for powerful predictive models will have to be established.

### Just-in-time adaptive intervention

**Fig. 2:**
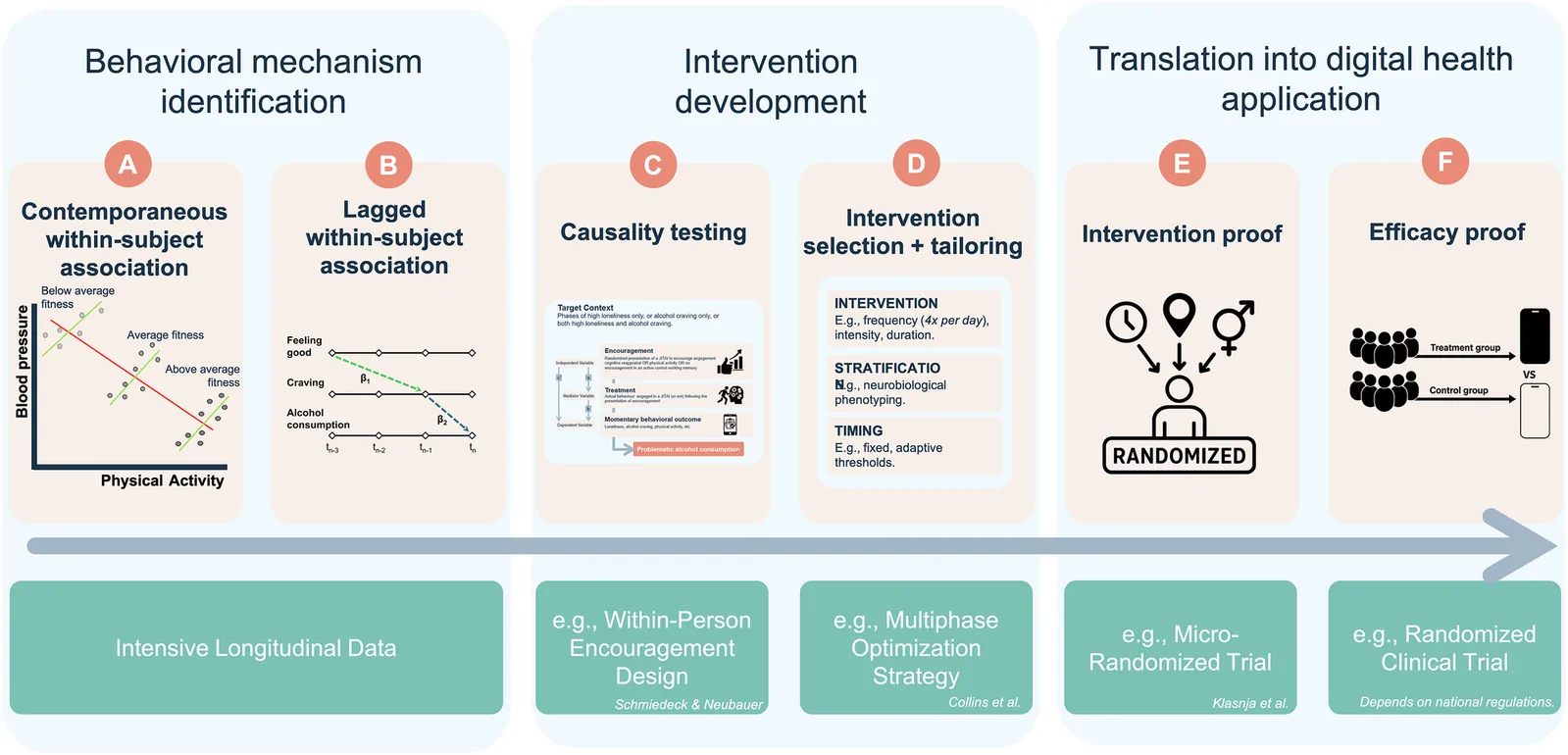
Methodological framework towards effective trialing of digital mental health interventions in daily life. The identification of behavioral mechanisms (left-hand panel) is based on intensive longitudinal data and offers insight into (**A**) contemporaneous within-subject association and (**B**) lagged within-subject association. This is complemented by intervention development (middle panel), which involves (**C**) causality testing and (**D**) intervention selection and tailoring through, for example, through within-person encouragement design or multiphase optimization strategy. The final step considers the translation into digital health applications. This involves (**E**) intervention proof (e.g., through micro-randomized trials) and (**F**) efficacy proof (e.g., through randomized control trials).

Just-in-time adaptive interventions are built upon the core assumption that daily life methods, such as e-diaries or wearables, can identify times and contexts in daily life when interventions are most needed and effective. In these moments, micro-interventions can readily be provided on wearable devices as patients go about their daily routines. However, to not intervene at unfavorable time points or to even trigger pathogenic mechanisms, daily life behavioral mechanisms of psychopathology must be understood in advance. For JITAIs to leverage their full potential, we here argue that this translates to the following requirements towards effective trialing of mental health interventions: (i) identification of within-subject behavioral mechanisms to establish causal inference under ecological validity to define intervention targets, (ii) development of suitable interventions by tailoring components to responders, times, and contexts, and testing efficacy in RCTs before, finally, (iii) translating them into digital health applications. As trial-and-error approaches are too costly, we propose a framework for efficiently targeting behavioral mechanisms. It describes research steps from between-subject findings towards digital interventions outlined below and illustrated in Fig. 2.

(i) Identification of behavioral mechanisms
  To improve mental health outcomes, mental health-promoting behaviors need to be repeated over time. For example, exercising, engaging in social interaction and mindfulness training can improve momentary mood states, but are not sufficient to treat a depressive episode. However, sustained engagement in these behaviors is effective in preventing and treating depression. Therefore, research towards expedient JITAIs needs to take a within-subject perspective. Importantly, within-person and between-person associations are distinct methodologically^42,43^, statistically^42,43^, and empirically^44–46^—a between-subject association cannot be used as a substitute for the within-subject process. These effects are independent—a phenomenon known as the ecological fallacy^47^. For example, as depicted in Fig. 2A, physically active individuals have lower habitual blood pressure, which translates to a negative between-subject correlation, although acute activity increases blood pressure within individuals, i.e., a positive within-subject correlation. For digital interventions, between-person associations are useful (e.g., for risk stratification) but cannot provide insights into within-subject mechanisms^48^.
(ii) Development of micro-interventions
  Observational within-person findings alone are insufficient as a basis for digital interventions. For instance, physical activity statistically predicts affective well-being in daily life, but also the reverse association has been shown^46^. Still, the causality of associations remains unknown. Causal inference requires: (1) correlation, (2) temporal order, (3) control of third variables, and (4) directionality^49^. Experimental designs allow causal testing under controlled conditions^50^. Experimental manipulations within everyday life^3^ offer a promising method towards causality testing within subjects using real-time data (e.g., e-diary ratings) and randomized intervention assignments (e.g., encouraging physical activity after low mood ratings^51^).
  However, experimental manipulation may be impractical in daily life due to ethical or logistical constraints; e.g., participants may be unable to follow intervention instructions under high work-related stress. The Within-Person Encouragement Design (WPED; Fig. 2C)^52^, addresses this via: (1) smartphone-based, randomly assigned encouragement to perform a micro-intervention as an instrumental variable (Encouragement), (2) micro-interventions (e.g., physical activity) themselves (Treatment), and (3) target variables (e.g., affective well-being) that are to be causally influenced by the Treatment (Outcome). Based on an instrumental variable approach, a causal treatment effect on the outcome can be analyzed if a correlation between the encouragement and the treatment is present and given the theoretical assumption that any encouragement effects on the outcome are fully mediated by the treatment (Fig. 2C). Indeed, pioneering studies applying the WPED design were able to reveal causal associations of sedentary breaks with affective well-being in everyday life^53^.
  Further, causal evidence alone does not determine optimal intervention dose or frequency. For example, to enhance well-being via physical activity, we must define: frequency (e.g., per day), intensity (e.g., light vs. moderate), duration (e.g., 10 vs. 30 minutes), type (e.g., stairs vs. jogging), and context (e.g., home, work) of the activity. To identify optimal micro-intervention delivery in everyday life, factorial analysis of variance (ANOVA) designs within the Multiphase Optimization Strategy (MOST; see Fig. 2D) can be used^54^. This enables to test different intervention components and their compositions against each other through randomizing intervention components between individuals. While ANOVA designs have been deemed impractical in traditional cross-sectional studies (a high number of conditions needed to be tested), they promise high value for JITAI development given their comparably easy implementation on mobile devices. Factorial designs are one of the most efficient approaches to simultaneously test the effect of different independent variables on an expected outcome. The gold-standard approach of a fully crossed factorial ANOVA can be reduced to a fractional ANOVA design, where it is not mandatory to include all possible intervention combinations, yet feasible to test only theoretically grounded subsets based on WPED results^54^.
  The way towards expedient digital mental health interventions involves tailoring the micro-interventions to time-points, places and contexts of greatest need and success. For instance, a physical activity intervention aimed at reducing negative mood and preventing alcohol consumption may be most effective during periods of heightened craving and in environments with drinking cues^55,56^. These intervention triggers and their effectiveness can vary significantly between individuals and may depend on neurobiological phenotypes—some patients may be vulnerable in social settings, others when alone. Such adaptive components should be simulated and tested. For example, in a physical activity intervention to improve psychological states, physical capacity may improve across time, in turn influencing the intervention efficacy. To account for such changes, intervention components can adjust activity levels based on individual progress. Decisions regarding timing, context, and adaptivity of the micro-intervention can be explored through simulations using intensive longitudinal data or tested experimentally, e.g., via the Sequential Multiple Assignment Randomized Trial (SMART) design within the MOST framework^54^ (see Fig. 2E).
(iii) Translation into digital health application.

Once behavioral targets are identified, causal inference under ecological validity is established, and interventions are tailored and proven effective, they can be translated into digital therapy. This step depends on regulatory frameworks of countries where the digital health application is deemed to be introduced on the market. It typically involves clinical trials using traditional randomized designs^57^ (see Fig. 2F).

### Summary

Digital interventions for mental health are promising, but have not yet lived up to expectations. Towards effective trialing of digital interventions, we here discussed potential flaws which might have caused gaps between promising pilot data and failing RCTs, namely implementation, alpha-inflation and unintentional temporal data leakage. In addition, we outlined specific proposals for future research: For just-in-time relapse prevention, we suggested that long-term investigations studying clinically well-characterized emerging episodes and including predictors of high validity are most promising for the development of powerful algorithms, which may later on prove efficacy in RTCs. Towards just-in-time adaptive interventions, we proposed the necessity of a within-subject perspective, known causal relations to intervene on, and basic knowledge on optimal frequency, intensity, duration, type and context of delivering interventions; our novel framework presented offers a schematic pathway towards effective trialing of digital mental mHealth interventions.

## Data Availability

No datasets were generated or analysed during the current study.

## References

[1] World Health Organization (WHO). *International Statistical Classification of Diseases and Related Health Problems (11th Revision; ICD-11)*. Available at https://icd.who.int/en (WHO, 2019).

[2] Serre, F. et al. The Craving-Manager smartphone app designed to diagnose substance use/addictive disorders, and manage craving and individual predictors of relapse: a study protocol for a multicenter randomized controlled trial. *Front. Psychiatry* **14**, 1143167 10.3389/fpsyt.2023.1143167 (2023). PMC10226427 37255691

[3] Trull, T. J. & Ebner-Priemer, U. Ambulatory assessment. *Annu. Rev. Clin. Psychol.* **9**, 151–176 10.1146/annurev-clinpsy-050212-185510 (2013). PMC4249763 23157450

[4] Müller, C. P. et al. Phase-sensitive individualized pharmacotherapy for alcohol use disorder. *Neuropharmacology* **283**, 110745 10.1016/j.neuropharm.2025.110745 (2026). 41151669

[5] Fried, E. I., Proppert, R. K. K. & Rieble, C. L. Building an early warning system for depression: rationale, objectives, and methods of the WARN-D study. *Clin. Psychol. Eur.* **5**, e10075 10.32872/cpe.10075 (2023). PMC10863640 38356901

[6] Wigman, J. T. W. et al. Digital health technologies in the accelerating medicines Partnership® Schizophrenia Program. *Schizophrenia* **11**, 83 10.1038/s41537-025-00599-w (2025). PMC12134270 40461469

[7] Larsen, M. E. et al. Using science to sell apps: Evaluation of mental health app store quality claims. *npj Digit. Med.* **2**, 18 10.1038/s41746-019-0093-1 (2019). PMC6550255 31304366

[8] Torous, J. A path towards progress: lessons from the hard things about digital mental health. *World Psychiatry* **21**, 419–420 10.1002/wps.21003 (2022). PMC9453910 36073703

[9] Anmella, G. et al. Smartphone-based interventions in bipolar disorder: systematic review and meta-analyses of efficacy. A position paper from the International Society for Bipolar Disorders (ISBD) Big Data Task Force. *Bipolar Disord.* **24**, 580–614 10.1111/bdi.13243 (2022). PMC9804696 35839276

[10] Ebner-Priemer, U. & Santangelo, P. Digital phenotyping: hype or hope? * Lancet Psychiatry* **7**, 297–299 10.1016/S2215-0366(19)30380-3 (2020). 31635970

[11] Smyth, J. M. & Ebner-Priemer, U. Dispelling “pleasing myths” about the integration of ecological momentary assessment and intervention into clinical research and practice. *World Psychiatry* **24**, 2–3 10.1002/wps.21262 (2025). PMC11733460 39810661

[12] Faurholt-Jepsen, M. et al. Daily electronic self-monitoring in bipolar disorder using smartphones - the MONARCA I trial: a randomized, placebo-controlled, single-blind, parallel group trial. *Psychol. Med.* **45**, 2691–2704 10.1017/S0033291715000410 (2015). 26220802

[13] Faurholt-Jepsen, M. et al. The effect of smartphone-based monitoring on illness activity in bipolar disorder: the MONARCA II randomized controlled single-blinded trial. *Psychol. Med.* **50**, 838–848 10.1017/S0033291719000710 (2020). 30944054

[14] Linardon, J. & Fuller-Tyszkiewicz, M. Attrition and adherence in smartphone-delivered interventions for mental health problems: a systematic and meta-analytic review. *J. Consult. Clin. Psychol.* **88**, 1–13 10.1037/ccp0000459 (2020). 31697093

[15] Liu, C., Messer, M., Linardon, J. & Fuller-Tyszkiewicz, M. Applying models of self-regulated learning to understand engagement with digital health interventions: a narrative review. *Front. Digit. Health* **7**, 1380088 10.3389/fdgth.2025.1380088 (2025). PMC12098494 40416095

[16] Deeken, F. et al. Patterns of alcohol consumption among individuals with alcohol use disorder during the COVID-19 pandemic and lockdowns in Germany. *JAMA Netw. Open* **5**, e2224641 10.1001/jamanetworkopen.2022.24641 (2022). PMC9344361 35913741

[17] Ebner-Priemer, U. W. et al. Trajectories of affective disorders: neurobiological mechanisms during symptom change. *Der Nervenarzt* **97**, 138–146 10.1007/s00115-025-01917-4 (2026). PMC12953256 41263959

[18] Daemen, M. et al. Efficacy of a transdiagnostic ecological momentary intervention for improving self-esteem (SELFIE) in youth exposed to childhood adversity: study protocol for a multi-center randomized controlled trial. *Trials* **22**, 641 10.1186/s13063-021-05585-y (2021). PMC8449997 34538272

[19] Reininghaus, U. et al. Transdiagnostic ecological momentary intervention for improving self-esteem in youth exposed to childhood adversity: the SELFIE randomized clinical trial. *JAMA Psychiatry* **81**, 227–239 10.1001/jamapsychiatry.2023.4590 (2024). PMC10687716 38019495

[20] Wang, T. et al. The Impact of gamification-induced users’ feelings on the continued use of mhealth apps: a structural equation model with the self-determination theory approach. *J. Med. Internet Res.* **23** e24546, 10.2196/24546 (2021). PMC8391751 34387550

[21] Eyben, F., Wöllmer, M. & Schuller, B. openSMILE: the Munich versatile and fast open-source audio feature extractor. In *Proc. 18th ACM International Conference on Multimedia* (eds Del Bimbo, A., Chang, S.-F. & Smeulders A.), 1459–1462 (ACM, 2010).

[22] Ebner-Priemer, U. W. et al. Digital phenotyping: towards replicable findings with comprehensive assessments and integrative models in bipolar disorders. *Int. J. bipolar Disord.* **8**, 35 10.1186/s40345-020-00210-4 (2020). PMC7677415 33211262

[23] Ludwig, V. M. et al. Predicting depressive and manic episodes in patients with bipolar disorder using statistical process control methods on passive sensing data. *J. Psychopathol. Clin. Sci.* 971–981 10.1037/abn0001002 (2025). 40689911

[24] Ludwig, V. M. et al. Early warning signals of bipolar relapse: Investigating critical slowing down in smartphone data. *J. Affect. Disord.* **391**, 119972 10.1016/j.jad.2025.119972 (2025). 40738339

[25] Davis, S. E., Matheny, M. E., Balu, S. & Sendak, M. P. A framework for understanding label leakage in machine learning for health care. *J. Am. Med. Inform. Assoc.* **31**, 274–280 10.1093/jamia/ocad178 (2024). PMC10746313 37669138

[26] Reichert, M. et al. Ambulatory assessment for precision psychiatry: foundations, current developments and future avenues. *Exp. Neurol.* **345**, 113807 10.1016/j.expneurol.2021.113807 (2021). 34228998

[27] Bolger, N. & Laurenceau, J.-P. *Intensive Longitudinal Methods. An Introduction to Diary and Experience Sampling Research* (Guilford Press, 2013).

[28] Wadle, L.-M. & Ebner-Priemer, U. W. Smart digital phenotyping. *Eur. Neuropsychopharmacol.* **76**, 1–2 10.1016/j.euroneuro.2023.07.002 (2023). 37451161

[29] Helmich, M. A. et al. Slow down and be critical before using early warning signals in psychopathology. *Nat. Rev. Psychol.* **3**, 767–780 10.1038/s44159-024-00369-y (2024).

[30] Mühlbauer, E. et al. Effectiveness of smartphone-based ambulatory assessment (SBAA-BD) including a predicting system for upcoming episodes in the long-term treatment of patients with bipolar disorders: study protocol for a randomized controlled single-blind trial. *BMC Psychiatry* **18**, 349 10.1186/s12888-018-1929-y (2018). PMC6204033 30367608

[31] Heinz, A. et al. Addiction Research Consortium: Losing and regaining control over drug intake (ReCoDe)-from trajectories to mechanisms and interventions. *Addictn. Biol.* **25**, e12866 10.1111/adb.12866 (2020). 31859437

[32] Matcham, F. et al. Remote assessment of disease and relapse in major depressive disorder (RADAR-MDD): a multi-centre prospective cohort study protocol. *BMC Psychiatry* **19**, 72 10.1186/s12888-019-2049-z (2019). PMC6379954 30777041

[33] Kircher, T. et al. Sonderforschungsbereich SFB/TRR 393: Verlaufsformen affektiver Erkrankungen: kognitiv-emotionale Mechanismen von Symptomveränderungen. *Der Nervenarzt*10.1007/s00115-025-01886-8 (2025). PMC12953339 40940529

[34] First, M. B., Williams, J. B. W., Karg, R. S. & Spitzer, R. L. *Structured Clinical Interview for DSM-5 Disorders (SCID-5)* (American Psychiatric Association, 2015).

[35] Montgomery, S. A. & Asberg, M. A new depression scale designed to be sensitive to change. *Br. J. Psychiatry: J. Ment. Sci*. **134**, 382–389 10.1192/bjp.134.4.382 (1979). 444788

[36] Matcham, F. et al. Remote Assessment of Disease and Relapse in Major Depressive Disorder (RADAR-MDD): recruitment, retention, and data availability in a longitudinal remote measurement study. *BMC Psychiatry* **22**, 136 10.1186/s12888-022-03753-1 (2022). PMC8860359 35189842

[37] Zech, H. G. et al. Mobile data collection of cognitive-behavioral tasks in substance use disorders: Where are we now? *Neuropsychobiology* **81**, 438–450 10.1159/000523697 (2022). 35350031

[38] Allen, K. et al. Using games to understand the mind. *Nat. Hum. Behav.* **8**, 1035–1043 10.1038/s41562-024-01878-9 (2024). 38907029

[39] King. *Candy Crush Saga [mobile application*] (Activision, 2012).

[40] Zech, H. et al. Measuring self-regulation in everyday life: Reliability and validity of smartphone-based experiments in alcohol use disorder. *Behav. Res. Methods* **55**, 4329–4342 10.3758/s13428-022-02019-8 (2023). PMC10700450 36508108

[41] Hachenberger, J. et al. Within-subject reliability, occasion specificity, and validity of fluctuations of the Stroop and go/no-go tasks in ecological momentary assessment. *Behav. Res. Methods* **57**, 29 10.3758/s13428-024-02567-1 (2024). PMC11682018 39733220

[42] Molenaar, P. C. M. On the implications of the classical ergodic theorems: analysis of developmental processes has to focus on intra-individual variation. *Dev. Psychobiol.* **50**, 60–69 10.1002/dev.20262 (2008). 18085558

[43] Zawadzki, M. J., Smyth, J. M., Sliwinski, M. J., Ruiz, J. M. & Gerin, W. Revisiting the lack of association between affect and physiology: Contrasting between-person and within-person analyses. *Health Psychol. Off. J. Div. Health Psychol. Am. Psychol. Assoc.* **36**, 811–818 10.1037/hea0000466 (2017). 28277697

[44] Kamarck, T. W., Schwartz, J. E., Janicki, D. L., Shiffman, S. & Raynor, D. A. Correspondence between laboratory and ambulatory measures of cardiovascular reactivity: a multilevel modeling approach. *Psychophysiology* **40**, 675–683 10.1111/1469-8986.00069 (2003). 14696722

[45] Reichert, M. et al. The association of stress and physical activity: mind the ecological fallacy. *Ger. J. Exerc. Sport Res.* **52**, 282–289 10.1007/s12662-022-00823-0 (2022).

[46] Timm, I., Giurgiu, M., Ebner-Priemer, U. & Reichert, M. The within-subject association of physical behavior and affective well-being in everyday life: a systematic literature review. *Sports Med.* **54**, 1667–1705 10.1007/s40279-024-02016-1 (2024). PMC11239742 38705972

[47] Robinson, W. S. Ecological correlations and the behavior of individuals. *Am. Sociol. Rev.* **15**, 351 10.2307/2087176 (1950).

[48] Meyer-Lindenberg, A. The non-ergodic nature of mental health and psychiatric disorders: implications for biomarker and diagnostic research. *World Psychiatry* **22**, 272–274 10.1002/wps.21086 (2023). PMC10168159 37159352

[49] Susser, M. What is a cause and how do we know one? A grammar for pragmatic epidemiology. *Am. J. Epidemiol.* **133**, 635–648 10.1093/oxfordjournals.aje.a115939 (1991). 2018019

[50] Gianicolo, E. A. L., Eichler, M., Muensterer, O., Strauch, K. & Blettner, M. Methods for evaluating causality in observational studies. *Dtsch. Arzteblatt Int.* **116**, 101–107 10.3238/arztebl.2020.0101 (2020). PMC7081045 32164822

[51] Klasnja, P. et al. Microrandomized trials: an experimental design for developing just-in-time adaptive interventions. *Health Psychol.* **34S**, 1220–1228 10.1037/hea0000305 (2015). PMC4732571 26651463

[52] Schmiedek, F. & Neubauer, A. B. Experiments in the wild: introducing the within-person encouragement design. *Multivar. Behav. Res.* **55**, 256–276 10.1080/00273171.2019.1627660 (2020). 31264902

[53] Giurgiu, M., Timm, I., Ebner-Priemer, U. W., Schmiedek, F. & Neubauer, A. B. Causal effects of sedentary breaks on affective and cognitive parameters in daily life: a within-person encouragement design. *npj Ment. Health Res.* **3**, 64 10.1038/s44184-024-00113-7 (2024). PMC11662072 39706901

[54] Collins, L. M., Murphy, S. A. & Strecher, V. The multiphase optimization strategy (MOST) and the sequential multiple assignment randomized trial (SMART): new methods for more potent eHealth interventions. *Am. J. Prevent. Med.* **32**, S112–S118 10.1016/j.amepre.2007.01.022 (2007). PMC2062525 17466815

[55] Serre, F., Fatseas, M., Swendsen, J. & Auriacombe, M. Ecological momentary assessment in the investigation of craving and substance use in daily life: a systematic review. *Drug Alcohol Depend.* **148**, 1–20 10.1016/j.drugalcdep.2014.12.024 (2015). 25637078

[56] Fatseas, M. et al. Craving and substance use among patients with alcohol, tobacco, cannabis or heroin addiction: a comparison of substance- and person-specific cues. *Addiction* **110**, 1035–1042 10.1111/add.12882 (2015). 25688760

[57] Federal Institute for Drugs and Medical Devices (BfArM). The Fast-Track Process for Digital Health Applications (DiGA) according to Section 139e SGB V. A Guide for Manufacturers, Service Providers and Users. Available at https://www.bfarm.de/SharedDocs/Downloads/EN/MedicalDevices/DiGA_Guide.html (2020).

